# Apolipoprotein E ε4 Allele-Based Differences in Brain Volumes Are Largely Uniform Across Late Middle Aged and Older Hispanic/Latino- and Non-Hispanic/Latino Whites Without Dementia

**DOI:** 10.3389/fnagi.2021.627322

**Published:** 2021-02-26

**Authors:** Ariana M. Stickel, Andrew C. McKinnon, Stephanie Matijevic, Matthew D. Grilli, John Ruiz, Lee Ryan

**Affiliations:** ^1^Department of Psychology, University of Arizona, Tucson, AZ, United States; ^2^Department of Neurosciences, University of California, San Diego, La Jolla, CA, United States; ^3^Brain and Mind Centre, University of Sydney, Camperdown, NSW, Australia

**Keywords:** apolipoprotein E ε4 allele, aging, brain, Latinos (U.S.), Hispanics, brain volume, cognition

## Abstract

Hispanics/Latinos are at an equal or a greater risk for Alzheimer's disease (AD), yet risk factors remain more poorly characterized as compared to non-Hispanic/Latino Whites. Among non-Hispanic/Latino White cohorts, the apolipoprotein E (APOE) ε4 allele is one of the strongest risk factors for AD with subtle declines in episodic memory and brain volumes detectable in the preclinical stages. We examined whether the APOE ε4 status had a differential impact on cognition and brain volumes among cognitively healthy and mild cognitively impaired Hispanics/Latinos (*n* = 86; ε4 *n* = 23) compared to a well-matched group of non-Hispanic/Latino Whites (*n* = 92; ε4 *n* = 29). Neither the APOE ε4 status nor the interaction between the ε4 status and ethnicity was associated with cognitive performance. The APOE ε4 status was associated with white matter and not with gray matter volumes. APOE ε4 carriers had a significantly smaller total brain white matter volumes, as well as smaller right middle temporal and left superior temporal volumes. The Hispanics/Latinos had significantly smaller left middle frontal gray matter volumes, yet marginally larger overall white matter volumes, than the non-Hispanic/Latino Whites. Exploratory analysis within the Hispanic/Latino sample found that those people whose primary language was Spanish had larger total brain white matter volumes compared primarily to the English speakers. Importantly, primary language differences only held for Hispanic/Latino ε4 carriers and did not differentiate Hispanic/Latino non-carriers, underscoring the need for further investigation into the impacts of language and acculturation on cognitive aging among the fastest growing ethnic minority group in the United States.

## Introduction

Hispanics/Latinos appear to be at least as likely as non-Hispanic/Latino Whites to develop Alzheimer's disease (AD) (Gurland et al., 1999; Haan et al., 2003), although estimated prevalence rates vary widely across studies. The Alzheimer's Association (2019) estimates a 1.5 times increased likelihood of developing the disease among Hispanics/Latinos compared to non-Hispanic/Latino Whites. The differences across studies may be due to the specific Hispanic/Latino country of origins studied [e.g., Mexican Americans (Haan et al., 2003) vs. Caribbean Hispanics/Latinos (Gurland et al., 1999)] or may be related to differences in how Hispanics/Latinos are impacted by risk factors for the disease and the degree to which such factors are considered in prevalence studies.

One of the largest risk factors for AD among non-Hispanic/Latino Whites is the apolipoprotein E (APOE) ε4 allele (Bertram et al., 2007). In the brain, the apolipoprotein E transports cholesterol and plays a role in neuronal repair (Villeneuve et al., 2014). Of the three allelic variants of the APOE gene (ε2, ε3, and ε4), the ε4 variant increases the risk for AD. Among non-Hispanic/Latino Whites, one copy of the ε4 allele is associated with a 3-fold increase in AD risk, and two copies of the ε4 allele are associated with a 15-fold increase in risk (Farrer et al., 1997). The APOE ε4 status impacts cognition, even among cognitively healthy (non-demented) older adults, perhaps as a prodrome of the disease (Foster et al., 2013) or perhaps independent of the disease (Greenwood and Parasuraman, 2003). Among cognitively healthy individuals, compared to non-carriers, APOE ε4 carriers have lower episodic memory scores in late middle age and older adulthood (Caselli et al., 2009) and lower scores on the Mini-Mental State Examination (MMSE), a measure of global cognition (Deary et al., 2002; Winnock et al., 2002). According to a meta-analysis (Small et al., 2004), APOE ε4 carriers have worse performance on episodic memory and executive functioning tasks in addition to having lower global cognition scores relative to non-carriers.

The APOE ε4 status is thought to impact cognition *via* its impact on brain structure and function. The APOE ε4 status has been associated with both increases (Han et al., 2007) and decreases (Filippini et al., 2011) in functional MRI activation while performing encoding tasks. Additionally, MRI deactivation during fixation relative to externally focused tasks was smaller for ε4 carriers compared to non-carriers (Pihlajamäki and Sperling, 2009). Data from PET suggest that compared to cognitively healthy ε4 non-carriers, the ε4 carriers show greater declines in brain metabolism within the temporal, prefrontal, posterior cingulate, parahippocampal, thalamic, and basal forebrain regions over a 2-year period (Reiman et al., 2001). The connections between the brain structure and cognitive performance are also influenced by the APOE ε4 status. For example, Wang et al. (2019) studied the relationship between the hippocampal volume and the long delay verbal memory scores among ε4 carriers and non-carriers with mild cognitive impairment (MCI). Among individuals with relatively small hippocampal volumes, ε4 carriers had lower performance on delayed verbal memory compared to non-carriers but the ε4 status did not differentiate memory performance among individuals with larger hippocampal volumes. Hippocampal atrophy is one of the earliest biomarkers of AD, and the APOE ε4 status has been associated with smaller hippocampi among cognitively healthy middle-aged and older adults (den Heijer et al., 2002; Taylor et al., 2014).

The impact of APOE ε4 status on the risk for AD among Hispanics/Latinos is largely unknown. Hispanics/Latinos are generally less likely to be carriers of the APOE ε4 allele than non-Hispanic/Latino Whites (Campos et al., 2013; Qian et al., 2017). In a study which combined data across more than 40 studies of older adults with and without AD (Farrer et al., 1997), ~15% of Hispanics/Latinos carried at least one ε4 allele compared to 24% of non-Hispanic/Latino Whites. Additionally, estimated frequencies of APOE ε4 carriers among individuals with a diagnosis of AD are mixed. Some studies find similar rates of ε4 carriers among Hispanics/Latinos and non-Hispanic/Latino Whites with a diagnosis of AD (Harwood et al., 2004), while others find lower rates of ε4 carriers among Hispanics/Latinos relative to non-Hispanic/Latino Whites (Farrer et al., 1997; Haan et al., 2003). Still, other studies find no increased risk for developing AD associated with ε4 among Hispanics/Latinos (Tang et al., 1998). For example, Farrer et al. (1997) reported that, among patients with AD, ~37% of non-Hispanic/Latino Whites carried at least one ε4 allele compared to ~19% among Hispanics/Latinos (which is close to the population base-rate of 15% among Hispanics/Latinos reported by Farrer et al., 1997). In contrast, when Hispanics/Latinos with AD, who identified their race as White, were compared to non-Hispanic/Latino Whites, the groups had equivalent rates (27%) of ε4 carriers (Harwood et al., 2004).

Hispanic/Latino country of origin may contribute to the difference in the prevalence of the APOE ε4 status and its influence on the risk for AD. In a recent analysis of over 10,000 Hispanic/Latino Americans genotyped for the APOE ε4 status, those from the Dominican Republic had the highest rates of ε4 carriers (at least one ε4 copy; 17.5%), while those from Mexico, Central America, and South America had lower rates, closer to 11% (González et al., 2018). In the same cohort, the APOE ε4 status was not associated with MCI prevalence, and this was consistent across the country of origin groups (González et al., 2019). Among a unique group of predominantly (77%) Cuban Hispanics/Latinos with AD, who identified their race as White, whose primary language was Spanish, and who were born outside of the United States, having at least one copy of the ε4 allele was linked to an earlier age of onset compared to non-carriers (Harwood et al., 2004). The earlier onset was comparable to that of non-Hispanic/Latino White ε4 carriers, but this association was statistically more robust among non-Hispanic/Latino Whites. In a study on Mexican Americans and non-Hispanic/Latino Whites comprising 28 individuals with AD and 28 cognitively healthy people in each ethnic group (overall *N* = 112), odds of developing AD based on APOE ε4 status differed by ethnicity (Campos et al., 2013). Among Mexican Americans, the presence of one or more ε4 allele made up ~32% of individuals in the group with AD and 25% of the cognitively healthy group vs. ~61 and 36% of non-Hispanic/Latino Whites who had AD or were cognitively healthy, respectively. Non-Hispanic/Latino Whites with at least one ε4 allele were at an increased risk for developing AD, controlling for age, sex, and education, while Mexican Americans with at least one ε4 allele were not at an increased risk for AD once demographic variables were taken into account. Similarly, in a larger study of 267 Mexican-Americans genotyped for APOE ε4 (O'Bryant et al., 2013), having at least one copy of the ε4 allele was not related to the increased odds of having cognitive impairment (either AD or MCI). When cognition was examined among the combined diagnostic groups (i.e., cognitively healthy, MCI, and AD), the APOE ε4 status was not associated with global cognitive functioning [based on (MMSE)] among Mexican Americans but was related to lower MMSE scores among non-Hispanic/Latino Whites. Instead, among both Mexican Americans and non-Hispanic/Latino Whites, having at least one ε4 allele was related to greater dementia symptom severity (O'Bryant et al., 2013) based on the Clinical Dementia Rating Scale (Morris, 1993).

Even less literature exists on the influence of the APOE ε4 status on the brain and the cognition among cognitively healthy Hispanics/Latinos. Consistent with studies among non-Hispanic/Latino Whites (Alexander et al., 2002), a study (Langbaum et al., 2010) on cognitively healthy middle-aged and older Hispanics/Latinos found that ε4 carriers had lower cerebral metabolism in the precuneus and posterior cingulate compared to non-carriers. Regarding the white matter and brain structure, among a racially diverse, predominantly Hispanic/Latino sample of individuals with a wide range of cognitive functioning, the APOE ε4 status was not associated with an increased risk for microbleeds in the brain (Caunca et al., 2016). However, among the same sample, ε4 carriers with higher total cholesterol had smaller white matter hyperintensity volumes, a marker of small vessel damage in the brain, compared to those with lower cholesterol, and this relationship was not significant among non-carriers (Willey et al., 2014).

Regarding cognition, among samples from 90-year-old Puerto Ricans without dementia, the ε4 status was associated with higher cognitive performance on a composite of visuospatial, naming, and attention tasks compared to non-carriers (Carrión-Baralt et al., 2009). Interestingly, the authors suggest that higher scores among ε4s may be attributable to a survivor effect; in other words, ε4 carriers who have survived up to their 90s may have other resilience factors offsetting the ε4 risk, or the ε4 status may actually be helpful among the oldest old (Carrión-Baralt et al., 2009). It is unknown if the APOE ε4 status is associated with higher or lower cognitive functioning performance among cognitively healthy Hispanics/Latinos in middle age and in earlier older adulthood.

Taken together, at present, the impact of the APOE ε4 status on aging Hispanics/Latinos is ambiguous, likely nuanced, and particularly understudied among cognitively healthy individuals. The present study examined whether ethnicity (i.e., Hispanic/Latino vs. non-Hispanic/Latino) modulates the relationships between the APOE ε4 status and (1) cognition (i.e., episodic memory, executive functioning, and processing speed) and (2) brain volumes among cognitively healthy older adults, aged from 50 to 94. We were particularly interested in the relationship between the hippocampal volume and the episodic memory performance. We predicted that ε4 carriers, regardless of ethnicity, would have lower episodic memory. Further, we predicted that this association would be stronger among non-Hispanic/Latino Whites than among Hispanics/Latinos. Similarly, we hypothesized that ε4 carriers would have smaller hippocampi than non-carriers and that this association would be stronger among non-Hispanic/Latino Whites compared to Hispanics/Latinos. In addition, we conducted a whole brain voxel-based morphometric analysis to determine whether other brain regions would be linked to the ε4 status, ethnicity, or the interaction between these two factors.

## Method

### Participants

The present study included 183 late middle-aged and older adults selected from the National Alzheimer's Coordinating Center (NACC)^1^ and the Alzheimer's disease neuroimaging initiative (ADNI) databases based on self-reported race and ethnicity and availability of neuroimaging and cognitive data. All participants provided informed consent. Data were collected in compliance with the Declaration of Helsinki. The NACC data used in this analysis were collected from 10 Alzheimer's Disease Research Centers from September 2005 to March 2016. The data used in the preparation of this article were also obtained from the ADNI database.^2^ The ADNI was launched in 2003 as a public–private partnership, led by the principal investigator, Michael W. Weiner, MD. The primary goal of ADNI has been to test whether serial MRI, PET, other biological markers, and clinical and neuropsychological assessment can be combined to measure the progression of MCI and early AD.^3^ We have previously published the data of 168 of these participants (see Stickel et al., 2019) in a study on cardiovascular disease risk factors and cognition. The present study included a total of 178 participants without a diagnosis of dementia, who at a minimum underwent neuroimaging and genotyping for APOE. The cohort included Hispanics/Latinos who self-identified their race as White (*n* = 86), in order to control the confounded race which may impact the strength of associations between risk factors and the brain structure and cognition (DeCarli et al., 2008; Glymour and Manly, 2008; Zahodne et al., 2015, 2017). Information about the country of origin was only available for 57 of the participants, with the majority (*n* = 47, 82.46%) of participants identifying Mexico as their country of origin. Baseline neuropsychological and neuroimaging examinations occurred between 2005 and 2015. Ethnic groups had age ranges from 50 to 94 years and were matched on several additional demographics. Ethnic groups, APOE ε4 groups, and the individual ethnicity by the APOE ε4 groups did not significantly differ in age, education, proportion of individuals with MCI, or apolipoprotein E ε4 status (ethnic group comparison only), systolic blood pressure, diastolic blood pressure, or body mass index (*F*s ≤ 2.04, n.s.; *t*s ≤ |1.33|, n.s.; χ^2^s ≤ 1.33, n.s.) (see Table 1).

**Table 1:** Combined Alzheimer's disease neuroimaging initiative (ADNI) and National Alzheimer's Coordinating Center (NACC) sample demographics organized by ethnicity and the APOE ε4 status.

	**Ethnicity**			
	**Hispanics/Latinos (*****n*** **=** **86)**		**Non-Hispanic/Latino Whites (*****n*** **=** **92)**	
	**ε4 non-carriers (*n* = 63)**	**ε4 carriers (*n* = 23)**	**ε4 non-carriers (*n* = 63)**	**ε4 carriers (*n* = 29)**
Age (years) *M* (±SEM)	72.03 (1.02)	71.87 (1.62)	72.22 (1.39)	68.90 (1.50)
Education (years) *M* (±SEM)	12.44 (0.62)	12.77 (0.80)	13.41 (0.37)	14.03 (0.47)
Systolic blood pressure (mm Hg) *M* (±SEM)	139.85 (2.38)	135.96 (4.53)	135.62 (2.82)	131.57 (2.89)
Diastolic blood pressure (mm Hg) *M* (±SEM)	72.84 (1.24)	75.30 (2.49)	74.70 (1.44)	73.82 (1.72)
Body mass index *M* (±SEM)	28.29 (0.67)	27.85 (0.96)	26.37 (0.54)	27.45 (1.02)
MCI (%)	44.44%	43.48%	42.86%	51.72%
Sex (% female)	61.90%	78.26%	60.32%	62.07%

*No significant group differences on any measures. APOE, Apolipoprotein; MCI, mild cognitive impairment*.

### Cognitive Measures

Participants in NACC and ADNI completed all or parts of the neuropsychological battery of the Uniform Data Set (Weintraub et al., 2009).^4^ The Uniform Data Set has multiple Spanish translations (Acevedo et al., 2009). There was no official Spanish translation of the Uniform Data Set from September 2005 to April 2007, and translations may have varied across Alzheimer's Disease Research Centers. Participants were tested in person and in their preferred language. In the present study, we included the following tests: the Wechsler Memory Scale (WMS) -III or -R Logical Memory Story delayed recall (Wechsler, 1987, 1997), the WAIS-R Digit Span Backward, the WAIS-R Digit Symbol (Wechsler, 1981), the Trail Making Test (Parts A and B; Reitan, 1958), and the MMSE (Folstein et al., 1975). ADNI participants were asked to take the Rey Auditory Verbal Learning Test (RAVLT; Rey, 1941) but neither the Story Recall nor the Digit-Symbol.

As described by Stickel et al. (2019), one memory measure was calculated across NACC and ADNI participants based on delayed recall on either a verbally presented story or a verbally presented list. Specifically, the Logical Memory Story IIA, recall (score range: 0–25), and the RAVLT (score range: 0–15) number correct raw scores were transformed to *z*-scores separately using the mean and SD of the corresponding NACC or ADNI sample. As a measure of executive functions, raw completion time on Trail B (score range: 0–300) was divided by a raw completion time on Trail A (score range: 0–150) to obtain a task switching measure (Trails B/A; Arbuthnott and Frank, 2000). Trail A requires that participants to draw lines to sequentially connect letters of the alphabet, and Trail B contains letters and numbers which need to be ordered sequentially while also switching back-and-forth between letters and numbers. The raw number of correct trials on Digit Span Backward (score range: 0–12) was a second measure of executive functions. It requires the participant to reverse the order of the verbally presented strings of numbers of increasing length maintained in the working memory. Trail A raw completion times and the raw number correct on the Digit-Symbol (score range: 0–93) were used as separate measures for processing speed. The Digit-Symbol requires individuals to transcribe symbols that correspond with a given number. We also included a general cognitive screening measure, the MMSE (score range: 0–30).

### Apolipoprotein E ε4 Status

To determine the APOE ε4 status, two single nucleotide polymorphisms (rs429358, rs7412) were analyzed to define the ε2, ε3, and ε4 alleles. The APOE ε4 status for NACC participants was obtained from individual NACC sites, the AD Genetics Consortium, and the National Cell Repository for Alzheimer's Disease (NCRAD). If the ε4 status of a participant differed between sources, then it was marked as missing.^5^ Specific genotyping methods for ADNI participants are described in Saykin et al. (2010). In the present study, the APOE ε4 status is defined as a carrier [someone who had one or two ε4 alleles (i.e., ε4 heterozygotes and homozygotes)] or a non-carrier (all others with no ε4 allele). Within the APOE ε4 carrier group, there were 47 (Hispanic *n* = 21) heterozygotes and 5 (Hispanic *n* = 2) homozygotes. Among APOE ε4 heterozygotes, 45 (Hispanic *n* = 21) carried a ε3 allele and 2 (no Hispanics) carried a ε2 allele. Given the small numbers of APOE ε4 homozygotes and ε2/ε4 heterozygotes, we were unable to compare specific ε4 allelic combinations.

### Image Acquisition

Participants underwent MRI collected on 1.5T and 3T scanners. T1 high-resolution images were collected from all participants included in the present study. The NACC neuroimaging data were collected using various standard clinical and research protocols available from multiple Alzheimer's Disease Research Centers. The ADNI encourages standardization by pre-approving specific scanners at research sites and reimburses sites for scans completed on pre-approved scanners. Both NACC and ADNI MRI scans undergo quality control checks, which help exclude scans with artifacts and those showing structural abnormalities, making it feasible for scans to be combined across imaging platforms (Beekly et al., 2007; Jack et al., 2010; Guo et al., 2017).^6^ For NACC MRI scans, quality control checks occur at the individual Alzheimer's Disease Research Center and then the de-identified MRI DICOM files were voluntarily submitted to NACC. Therefore, motion and coverage artifacts may occasionally be present despite the efforts by Alzheimer's Disease Research Center to avoid their inclusion and may not be screened out upon submission to NACC.

### Image Processing

Raw images were downloaded from the NACC and ADNI databases. Images were reconstructed and a non-brain tissue was removed using FreeSurfer v5.3 (Dale et al., 1999).^7^ Next, voxel-based morphometry (VBM) (Ashburner, 2007) was performed using the Diffeomorphic Anatomical Registration through Exponentiated Lie algebra (DARTEL) (Ashburner, 2007) in SPM8, a statistical parametric mapping.^8^ More specifically, whole brain volumes were aligned along the anterior to posterior commissural plane. Aligned images were then re-sliced to 1 mm^3^ and segmented into gray matter, white matter, and cerebrospinal fluid using tissue probability maps obtained from the VBM toolbox. Intracranial volumes (ICV) were calculated based on the summation of gray matter, white matter, and cerebrospinal fluid volumes. ICV was used to control the head size in all subsequent analyses of total and regional volumes. Additionally, the total gray matter and the total white matter volumes extracted in segmentation were used as total volumetric measures.

For the VBM analysis, DARTEL was used to create two study-specific custom templates for gray and white matters from the segmented gray and white matter images of all participants, respectively. The gray and white matter images of each participant were then normalized to the appropriate study-specific template, modulated using Jacobian determinants, and smoothed with a 10 mm full-width-half-maximum (FWHM) isotropic Gaussian kernel.

Two multiple regression analyses (one for gray matter, one for white matter) were performed in SPM8, in order to identify the regions of the brain in which the impact of the APOE ε4 status was moderated by ethnicity. Regressions included the APOE ε4 status and ethnicity as main exposures and the interaction between the APOE ε4 status and ethnicity, as well as age and ICV, as the control variables. Initially, the statistical threshold cluster for each association tested was set at *p* < 0.05, with the corrected false discovery rate (FDR) (Benjamini and Hochberg, 1995). However, no voxels met this criterion. The threshold was then changed at *p* < 0.001, uncorrected, with a cluster of 50 contiguous voxels. This threshold was chosen based on a previous study that tested the impact of a genetic risk factor for cognitive impairment on brain volumes; see Stickel et al. (2018). The cluster threshold was more conservative than previous VBM analyses from our laboratory using the same *p*-threshold and a cluster size of 18, which was determined based on Monte Carlo simulations (Walther et al., 2011), but was less conservative than some other VBM analyses using the same *p*-value (Glodzik et al., 2012: k = 75; ten Kate et al., 2016: k = 100). MarsBaR (Brett et al., 2002) was used to extract volumetric values per person for each significant cluster identified in the VBM analysis. Volumes were calculated as the average value across all significant voxels within a given cluster.

Finally, given that we were also interested in the hippocampal volumes, the Wake Forest University PickAtlas 2.0 (Maldjian et al., 2003) and Automated Anatomical Labeling library (Tzourio-Mazoyer et al., 2002) were used to create a mask of the left and the right hippocampi that was applied to the gray matter map of each individual in MarsBaR in order to extract the hippocampal volumes. Regional and total volumes as well as ICV values for each person were entered into SPSS v 25 (IBM Corp, released 2017, Armonk, NY, USA). Once entered into SPSS, all volumes were residualized for ICV prior to conducting further analyses.

### Statistical Analysis

In order to test the hypothesis that the APOE ε4 status is related to cognition differentially between the two ethnic groups, five [one per cognitive task (i.e., delayed verbal memory, Trails B/A, Digit Span Backward, Trails A, and Digit-Symbol)] general linear models (GLMs) were performed in SPSS v 25 (IBM Corp, released 2017, Armonk, NY, USA). Each GLM tested the independent impact of the APOE ε4 status and ethnicity, as well as the interaction between the APOE ε4 status and ethnicity, controlling for age and education.

Five additional GLMs were conducted to test the main associations between exposures (APOE ε4 status, ethnicity, and the interaction between APOE ε4 status and ethnicity) and total gray matter, total white matter, and the hippocampal (left, right, bilateral) volumes. Age was included as a covariate.

## Results

### APOE ε4, Ethnicity, and Cognition

Results shown in Table 2 are *F*-statistics from GLMs unless there was a violation of homoscedasticity, in which case robust parameter estimates (*t*-statistics) are reported. Overall models for delayed verbal memory, Digit Span Backward, Trail A, Digit-Symbol, and the MMSE were significant. Controlling for age and education, neither the APOE ε4 status nor the interaction between the APOE ε4 status and ethnicity was related to any cognitive measures (*F*s ≤ 2.62, n.s.; *t*s ≤ |1.24|, n.s.). Hispanics/Latinos had significantly lower scores on the Digit-Symbol [*F*_(1, 85)_ = 22.58, *p* < 0.001] and marginally lower performance on Digit Span Backward (*t* = 1.71, *p* = 0.09) compared to non-Hispanic/Latino Whites. Age and education covariates were each associated with performance on delayed verbal memory, Trail A, and Digit-Symbol (*F*s ≥ 4.08, *p* < 0.05; *t*s ≥ |3.13|, *p* < 0.01). Additionally, education was associated with scores on Digit Span Backward (*t* = 3.14, *p* < 0.01) and the MMSE (*t* = 4.05, *p* < 0.001).

**Table 2:** *F*-statistics for overall models (in bold) and exposure/covariate factors for models.

Delayed verbal memory	
Overall model	*F*_(5, 152)_ = 5.91***
APOE	*F*_(1, 152)_ = 2.03
Ethnicity	*F*_(1, 152)_ = 1.63
Age	*F*_(1, 152)_ = 19.27***
Education	*F*_(1, 152)_ = 4.08*
APOE × ethnicity	*F*_(1, 152)_ = 2.32
Digit span backward	
Overall model	*F*_(5, 117)_ = 7.01***
APOE	*t* = −0.71
Ethnicity	*t* = 1.71
Age	*t* = −0.51
Education	*t* = 3.14**
APOE × ethnicity	*t* = 0.12
Trails B/A	
Overall model	*F*_(5, 149)_ = 0.96
APOE	–
Ethnicity	–
Age	–
Education	–
APOE × ethnicity	–
Trail A	
Overall model	*F*_(5, 155)_ = 20.86***
APOE	*t* = 0.52
Ethnicity	*t* = −0.97
Age	*t* = 3.13**
Education	*t* = −5.44***
APOE × ethnicity	*t* = −1.24
Digit-symbol	
Overall model	*F*_(5, 85)_ = 25.78***
APOE	*F*_(1, 85)_ = 2.62
Ethnicity	*F*_(1, 85)_ = 22.58***
Age	*F*_(1, 85)_ = 15.14***
Education	*F*_(1, 85)_ = 48.85***
APOE × ethnicity	*F*_(1, 85)_ = 1.72
Mini-mental state examination	
Overall model	*F*_(5, 145)_ = 9.80***
APOE	*t* = 0.50
Ethnicity	*t* = 0.66
Age	*t* = −1.72^†^
Education	*t* = 4.05***
APOE × ethnicity	*t* = 0.66

t-statistics from robust standard errors are included in place of F-statistics for models in which there was a violation of homoskedasticity. p < 0.10, *p < 0.05, **p < 0.01, ***p < 0.001.

### APOE ε4, Ethnicity, and Total and Hippocampal Volumes

All volumes were residualized for ICV prior to being analyzed. Each GLM tested the independent associations of the APOE ε4 status and ethnicity, as well as the interaction between the APOE ε4 status and ethnicity on volumes, controlling for age. Overall models for total brain gray matter, total brain white matter, the left hippocampus, the right hippocampus, and the bilateral hippocampus were significant. Controlling for age, the ε4 carriers had significantly smaller total brain white matter volumes [*F*_(1, 173)_ = 4.18, *p* < 0.05] than non-carriers, and Hispanics/Latinos had marginally larger white matter volumes than non-Hispanic/Latino Whites [*F*_(1,173)_ = 3.24, *p* = 0.07]. Gray matter volumes and hippocampal volumes were not associated with the APOE ε4 status, ethnicity, or the APOE ε4 status by ethnicity interaction (*F*s ≤ 0.39, n.s.; *t*s ≤ |0.93|, n.s.). Older age was associated with smaller brain volumes in all brain measures (*F*s ≥ 17.41; *t* = −3.59), except total brain white matter volumes [*F*_(1, 173)_ = 2.73, n.s.]. See Table 3 for results.

**Table 3:** *F*-statistics for overall models (in bold) and exposure/covariate factors for models predicting total gray matter, total white matter, and hippocampal brain volumes.

Total brain gray matter	
Overall model	*F*_(4, 173)_ = 4.59**
APOE	*t* = −0.93
Ethnicity	*t* = 0.003
Age	*t* = −3.59***
APOE × ethnicity	*t* = 0.15
Total brain white matter	
Overall model	*F*_(4, 173)_ = 3.01*
APOE	*F*_(1, 173)_ = 4.18*
Ethnicity	*F*_(1, 173)_ = 3.24^†^
Age	*F*_(1, 173)_ = 2.73
APOE × ethnicity	*F*_(1, 173)_ = 0.11
Left hippocampus	
Overall model	*F*_(4, 173)_ = 4.94***
APOE	*F*_(1, 173)_ = 0.02
Ethnicity	*F*_(1, 173)_ = 0.20
Age	*F*_(1, 173)_ = 18.46***
APOE × ethnicity	*F*_(1, 173)_ = 0.39
Right hippocampus	
Overall model	*F*_(4, 173)_ = 4.63**
APOE	*F*_(1, 173)_ = 0.00003
Ethnicity	*F*_(1, 173)_ = 0.31
Age	*F*_(1, 173)_ = 17.41***
APOE × ethnicity	*F*_(1, 173)_ = 0.17
Bilateral hippocampus	
Overall model	*F*_(4, 173)_ = 4.91***
APOE	*F*_(1, 173)_ = 0.004
Ethnicity	*F*_(1, 173)_ = 0.26
Age	*F*_(1, 173)_ = 18.41***
APOE × ethnicity	*F*_(1, 173)_ = 0.28

All brain volumes residualized for intracranial volume prior to analysis. t-statistics from robust standard errors are included in the place of F-statistics for models, in which there was a violation of homoskedasticity. †p < 0.10, *p < 0.05, **p < 0.01, ***p < 0.001.

### Exploratory Analysis of Primary Language and APOE ε4 Status Among Hispanics/Latinos

**Figure 1:**
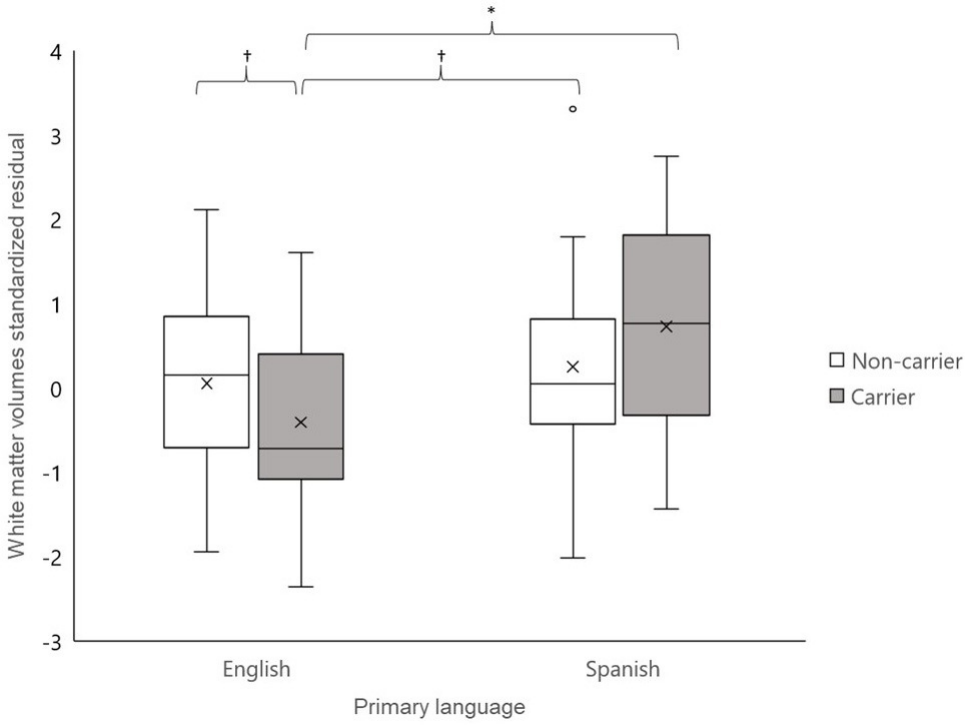
Total brain white matter volumes residualized for intracranial volume for Hispanics/Latinos whose primary language was English (left) and whose primary language was Spanish (right). Apolipoprotein E (APOE) ε4 carriers whose primary language was English had marginally to significantly smaller white matter volumes than the other three groups (*t*s ≥ |1.89|, *ps* < 0.07), controlling for age and education. No other group differences were detected (*t*s ≤ |1.20|, n.s.). ^†^*p* < 0.10; *p < 0.05.

Acculturation differences between Hispanics/Latinos have been shown to influence lifespan in the presence of certain diseases (Gallo et al., 2009). Greater adherence to Hispanic/Latino culture is associated with more resiliency, but the impact of acculturation on genetic risk for AD is unknown. In order to test if a factor related to acculturation, the primary language, may have influenced the impact of APOE ε4 status on total white matter volumes, we conducted an exploratory *post-hoc* analysis specific to the Hispanic/Latino sample. Briefly, Hispanics/Latinos with Spanish as their primary language (*n* = 37) did not differ from those whose primary language is English (*n* = 49) on age (*t* = 0.19, n.s), with cardiovascular health indicators [i.e., systolic blood pressure, diastolic blood pressure, body mass index (*F*s ≤ 1.60, n.s.)], from different sex, cognitive status, or history of hypertension (χ^2^s ≤ 2.71, n.s.), but they had lower education, on average (~11 vs. 14 years, *t* < 3.66, *p* < 0.01). Additionally, the primary Spanish language group had marginally fewer ε4 carriers than the primary English language group [$\chi_{\left(1\right)}^{2}$ = 3.67, *p* = 0.06] (Table 4). We conducted GLMs in SPSS, testing the relationships between total brain volumes and the APOE ε4 status, primary language, and the interaction of the APOE ε4 status and primary language, controlling for age and ICV. We also controlled for education, given that the language groups significantly differed on this variable. The interaction between the APOE ε4 status and primary language was significantly linked to total brain white matter volumes [*F*_(1, 77)_ = 4.04, *p* < 0.05]. Simple association tests determined that ε4 carriers who primarily spoke English had significantly smaller white matter volumes compared to ε4 carriers who primarily spoke Spanish (*t* = −2.09, *p* < 0.05) as well as marginally smaller volumes compared to both non-carrier groups (vs. Spanish: *t* = −1.89, *p* = 0.07; vs. English: *t* = −1.91, *p* = 0.06). No other differences in simple associations were detected (*t*s ≤ |1.20|, n.s.) (Figure 1). Older age (marginally) and lower education were also associated with larger white matter volumes [*F*_(1,77)_ = 3.26, *p* = 0.08 and *F*_(1,77)_ = 4.15, *p* < 0.05, respectively]. The main association between the APOE ε4 status and primary language were not associated with white matter volumes (*F* ≤ 2.04, n.s.).

**Table 4:** Participant demographics for Hispanics/Latinos organized by primary language and APOE ε4 status.

	**Primary language**			
	**English (*****n*** **=** **49)**		**Spanish (*****n*** **=** **37)**	
	**ε4 non-carriers (*n* = 32)**	**ε4 carriers (*n* = 17)**	**ε4 non-carriers (*n* = 31)**	**ε4 carriers (*n* = 6)**
Age (years) *M* (±SEM)	71.94 (1.51)	72.65 (2.03)	72.13 (1.40)	69.67 (2.38)
Education (years) *M* (±SEM)**	14.03 (0.52)	13.76 (0.53)	10.69 (1.09)	9.40 (2.68)
Systolic blood pressure (mmHg) *M* (±SEM)	140.81 (3.18)	134.18 (5.42)	138.83 (3.62)	141.00 (8.51)
Diastolic blood pressure (mmHg) *M* (±SEM)	72.84 (1.63)	74.12 (3.06)	72.83 (1.90)	78.67 (4.10)
Body mass index *M* (±SEM)	28.14 (1.05)	27.42 (1.26)	28.45 (0.84)	29.05 (0.93)
MCI (%)	37.50%	35.29%	51.61%	66.67%
Sex (% female)	59.38%	76.47%	64.52%	83.33%

APOE, Apolipoprotein; MCI, mild cognitive impairment. **p < 0.01.

### APOE ε4, Ethnicity, and Voxel-Based Morphometry Analysis

**Figure 2:**
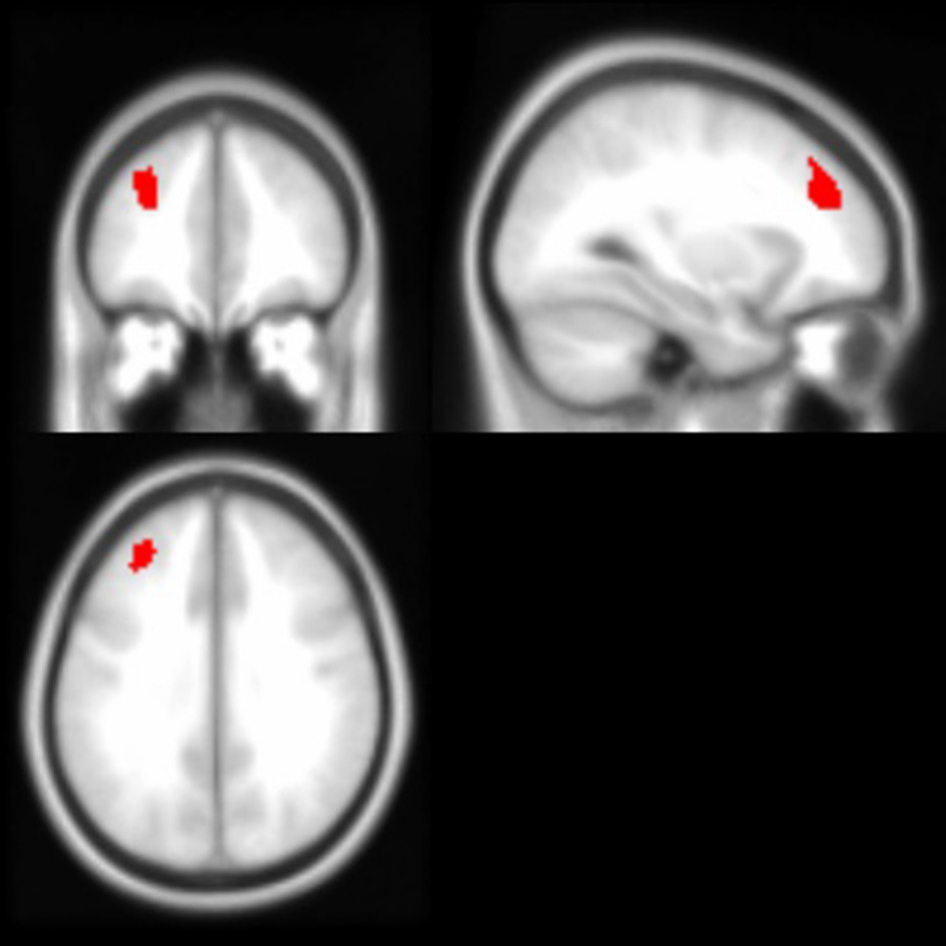
Brain images depicting the gray matter region in which non-Hispanic/Latino Whites had larger gray matter volumes compared to Hispanics/Latinos, controlling for age and intracranial volume (*p* < 0.001, k > 50). This region (in red) is in the left middle frontal gyrus; Montreal Neurological Institute (MNI) coordinates: −30, 40, 32.

**Figure 3:**
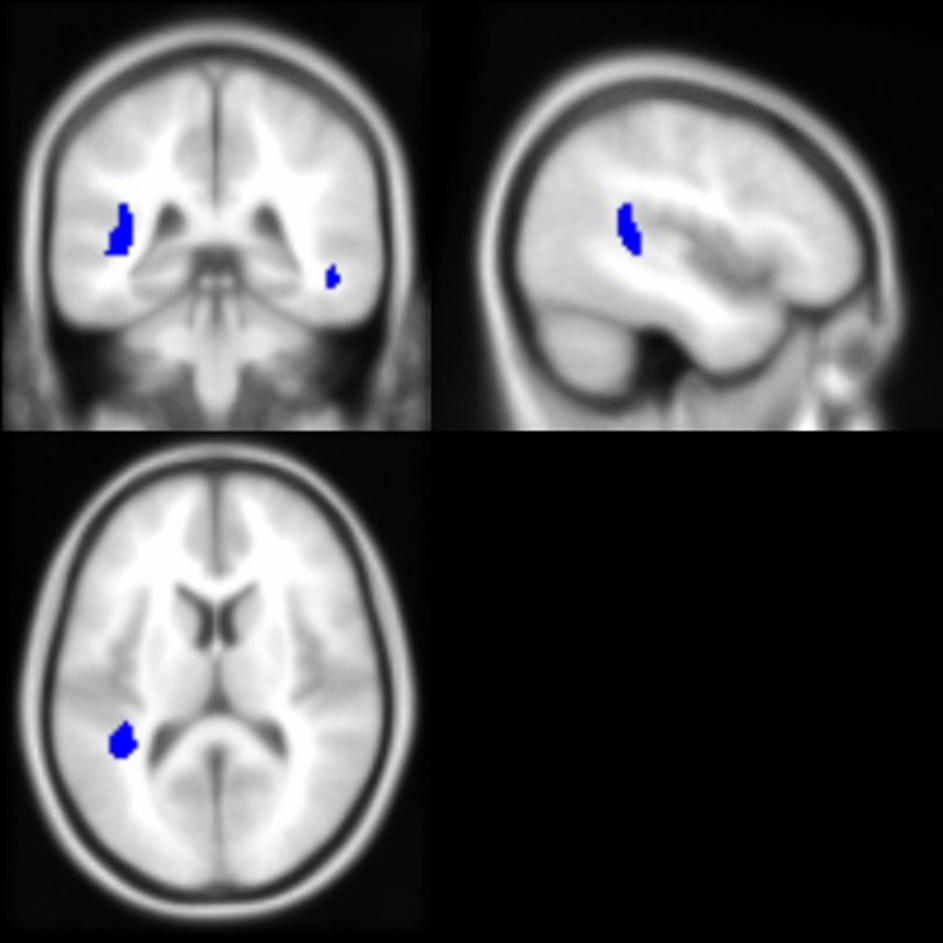
Brain images depicting white matter regions in which APOE ε4 carriers had smaller volumes compared to non-carriers, controlling for age and intracranial volume (*p* < 0.001, k > 50). These regions (in blue) are within the right middle temporal and left superior temporal white matter; MNI coordinates for depicted image slices: −44, −38, 12.

As noted above, the voxel-based morphometry (VBM) analysis tested the impact of the APOE ε4 status, ethnicity, and the interaction between the APOE ε4 status and ethnicity on each gray and white matter voxel in the brain. At a threshold of *p* < 0.001 and k = 50, one significant gray matter region was identified: non-Hispanic/Latino Whites had larger gray matter volumes in the left middle frontal gyrus compared to Hispanic/Latino individuals (Figure 2). Within the white matter, APOE ε4 carriers had smaller volumes in the left superior temporal white matter near the temporal parietal junction and in the right middle temporal lobe compared to non-carriers (Figure 3). See Table 5 for the cluster table. The APOE ε4 by ethnicity interaction was not associated with brain volumes.

**Table 5:** Regions in which group (ethnicity or APOE ε4 status) differences in volumes were detected, *p* < 0.001, k ≥ 50, controlling for age and intracranial volume.

	**MNI coordinates**				
**Region**	**x**	**y**	**z**	***t*-value (peak intensity)**	**Cluster size**
**Gray matter region**					
***Non-Hispanic/Latino Whites > Hispanics/Latinos***					
L middle frontal gyrus	−30	40	32	3.66	1,498
**White matter regions**					
***APOEε4 Non-carriers>carriers***					
R middle temporal	52	−38	−9	3.67	299
L superior temporal	−42	−43	15	3.99	2,649

*The Montreal Neurological Institute (MNI) coordinates of the location of maximal significance, the t-value, and cluster size (in mm^3^) of each cluster are provided. L, left hemisphere; R, right hemisphere*.

## Discussion

In our study on Hispanics/Latinos and non-Hispanic/Latino Whites, the APOE ε4 status was not associated with cognitive outcomes but was linked to brain volumes. APOE ε4 carriers had smaller white matter volumes overall (total brain) and within bilateral temporal regions compared to non-carriers across both ethnicities. Investigating the Hispanic/Latino sample further, we found that ε4 carriers who primarily spoke Spanish had larger total brain white matter volumes than those who primarily spoke English, whereas volumes among non-carriers were similar between language groups. Over and above the impact of the APOE ε4 status, Hispanics/Latinos had marginally larger total brain white matter volumes but significantly smaller gray matter volumes in the left middle frontal gyrus compared to non-Hispanic/Latino Whites. Our results suggest important biological commonalities between the ethnic groups while also highlighting the importance of examining the factors that are highly relevant and heterogeneous within the Hispanic/Latino population (e.g., language use, acculturation). Our findings are discussed in more detail in the following sections.

### Cognition

We did not detect an association between the APOE ε4 status and performance in any cognitive domain examined: episodic memory, executive functioning, and processing speed. Rather, the covariates of age and education accounted for the majority of the variance in cognition. In contrast to our predictions, the APOE ε4 status was not associated with episodic memory. This is in contrast to the majority of existing literature (Deary et al., 2002; Caselli et al., 2009). According to a meta-analysis (Small et al., 2004), lower performance on episodic memory, global cognition, and executive functioning are consistently found among APOE ε4 carriers relative to non-carriers.

There may be several reasons why we did not observe an association of APOE ε4 status and cognition in the present study. It may be that across the relatively wide age range (50–94 years) in the present study, the impact of APOE ε4 status may change with increasing age rather than having a uniform effect among all older adults. Genetic factors that influence cognition appear to have stronger impact in older age prior to the onset of dementia (Lindenberger et al., 2008; Papenberg et al., 2015; Stickel et al., 2018), possibly due to weakening structural integrity and/or impairments in neural processing. Supporting this notion, some have found that the presence of one or more APOE ε4 alleles is linked to poorer cognition, specifically among older adults (Rawle et al., 2018), and cognitive decline (as opposed to baseline cognition) may be more sensitive to differences in the APOE ε4 status (Li et al., 2019). In contrast, one of the few studies of APOE ε4 on Hispanics/Latinos found that ε4 carriers of 90 year olds had higher scores on visuospatial, attention, and naming tasks, as well as nominally higher memory performance than non-carriers (Carrión-Baralt et al., 2009). As noted earlier, this may reflect a survivor effect, which outweighs the negative impacts of APOE ε4, or may reflect a change from a harmful to a beneficial role of the APOE ε4 status among the oldest. Alternatively, genetic ancestry may influence the impact of the APOE ε4 status on Hispanic/Latino cognitive aging (Blue et al., 2019; Granot-Hershkovitz et al., 2020).

Recent data suggest that, first, greater Amerindian genetic ancestry is associated with protection against APOE ε4-related cognitive declines (Granot-Hershkovitz et al., 2020). Second, education may be interacting with APOE ε4 to impact episodic memory (Christensen et al., 2008) as well as to diminish the association of APOE ε4 and global cognition when it is taken into account (Winnock et al., 2002). Along with age and education, there may be other factors contributing to the extent to which the APOE ε4 status impacts episodic memory, such as the presence of amyloid beta (Lim et al., 2013, 2015). High cerebral amyloid beta load may be a sensitive indicator of risk for cognitive impairment, but only for certain groups. For example, higher rates of amyloid beta were correlated with lower performance on verbal memory, visual memory, and working memory to a significantly stronger extent among cognitively healthy older ε4 carriers compared to non-carriers (Lim et al., 2013). Similarly, existing evidence suggests that APOE ε4 carriers may be more vulnerable to poor cognition in the presence of white matter hyperintensities, an indicator of small vessel damage, than non-carriers (Mirza et al., 2019). Even less is known about the interactions between the APOE ε4 status and amyloid beta or cerebrovascular damage on cognition among Hispanics/Latinos.

In the present study, Hispanics/Latinos and non-Hispanic/Latino Whites differed on multiple cognitive measures as was expected, given the previous cognitive comparisons of the same sample (Stickel et al., 2019). We found lower performance on the measures of working memory and one of the two processing speed measures among Hispanics/Latinos compared to non-Hispanic/Latino Whites. Notably, these associations persist even when taking into account the APOE ε4 status, age, and education. In contrast to O'Bryant et al. (2013), the impact of ethnicity on cognition did not vary by the ε4 status. Given that the APOE ε4 status alone largely produced null findings in the current study, it is difficult to determine whether the role of the APOE ε4 status on cognition is uniform across the ethnic groups.

### Total Brain Volumes

The present study detected APOE ε4-related differences for total brain white matter volumes, but not gray matter volumes. Specifically, ε4 carriers had smaller total brain white matter volumes than non-carriers. Ready et al. (2011) found a similar pattern of smaller total brain white matter volumes among ε4 carriers compared to non-carriers, and smaller volumes were associated with lower cognitive performance (i.e., slower processing speed and lower inhibition). Given that larger white matter volumes are not always reflective of greater white matter health, we cannot say whether the current findings support a ε4 disadvantage. For example, one study (Walther et al., 2010) found that higher body mass index was associated with larger white matter volumes. However, in a second study investigating white matter integrity within the same regions that were larger in volume, the authors found lower white matter integrity among those with higher body mass index (Ryan and Walther, 2014). Further, lower white matter integrity was associated with lower memory, lower executive functioning, and processing speed. Given that the present study also detected marginally larger total brain white matter volumes among Hispanics/Latinos relative to non-Hispanic/Latino Whites, future investigations into white matter integrity among Hispanics/Latinos may help clarify whether this is a structural advantage or disadvantage.

Heise et al. (2011) also found the evidence of ε4-related differences in the white matter and not in the gray matter in cognition using diffusion imaging metrics reflecting the integrity of the white matter. Specifically, the authors found that ε4 carriers had similar white matter volumes but lower white matter integrity compared to non-carriers across both young and older adults. Ryan et al. (2011) also found that APOE ε4 carriers had greater age-related poorer white matter integrity compared to non-carriers across all white matter regions (including the temporal stem, the frontal, lateral, and parietal centrum semiovale and genu, and splenium white matter regions). In the same cohort, the APOE ε4 status was associated with memory indirectly *via* the temporal stem (McKinnon et al., 2021). That is, ε4 carriers had poorer white matter integrity in the temporal stem relative to non-carriers, which was then associated with poorer memory.

With respect to ethnicity, we found marginally larger total white matter volumes among Hispanics/Latinos compared to non-Hispanic/Latino Whites. This is consistent with larger total brain volumes among Hispanics/Latinos compared to African Americans and non-Hispanic/Latino Whites observed in the study of DeCarli et al. (2008). DeCarli et al. (2008) noted that larger brain volumes among Hispanics/Latinos were only apparent after taking into account the ICV and suggested that Hispanics/Latinos may actually have smaller heads premorbidly. In the present study, ICV was taken into account, so it is unlikely to be a confound.

Future studies need to investigate whether APOE ε4 status would have the same associations among more diverse samples of Hispanics/Latinos. Remarkably, Hispanics/Latinos with AD tend to live longer after diagnosis than their non-Hispanic/Latino White counterparts (Helzner et al., 2008). Such findings are consistent with the Hispanic/Latino mortality paradox, which is the robust finding to confirm that Hispanics/Latinos tend to outlive non-Hispanic/Latino Whites (Abraido-Lanza et al., 1999; Ruiz et al., 2013) despite having, on average, higher rates of poverty, less education, and less access to medical care. Hispanic/Latino cultural factors, such as valuing strong family ties and collectivism, have been posited to have protective effects on the lifespan of Hispanic/Latinos (Gallo et al., 2009). Given that the present Hispanic/Latino sample identified their race as White, they may be more genetically similar to non-Hispanic/Latino Whites than other groups of Hispanics/Latinos and may also be more acculturated than other Hispanic/Latino samples. Acculturation is a multifaceted concept, and the ADNI and NACC databases do not collect information to adequately measure acculturation, but some information on language preference, one of the many components of acculturation, is collected. The *post-hoc* analysis on the Hispanic/Latino sample found that ε4 carriers who primarily spoke Spanish had larger total brain white matter volumes than those who primarily spoke English. Among non-carriers, the primary language did not differentiate the white matter volumes. Taken together, this may point to a difference based on acculturation. However, larger sample sizes and more thorough measures of acculturation and language would be required to differentiate the possibility of an effect related to acculturation vs. bilingualism, which is also hypothesized to be protective of brain integrity and function (Grant et al., 2014). Additionally, as noted earlier, diffusion imaging would also be helpful in providing more information about the quality of the white matter.

### Hippocampal Volumes

Also contrary to expectations, the APOE ε4 status was not linked to hippocampal gray matter volumes although it was associated with volumes elsewhere in the temporal lobes. The APOE ε4 status has been associated with smaller hippocampi among cognitively healthy middle-aged and older adults in cross-sectional studies (den Heijer et al., 2002; Taylor et al., 2014). However, Jak et al. (2007) found that the APOE ε4 status was predictive of declines in hippocampal volumes over an average of 17 months but was not associated with cross-sectional volume differences. Perhaps the present sample would detect an impact of the APOE ε4 status on hippocampal volumes over time. Another possible contributor to null findings in the present study was the inclusion of cognitively healthy individuals. Khan et al. (2017) observed significantly smaller hippocampi among ε4 carriers compared to non-carriers with AD and MCI, but this association was only marginal among cognitively healthy older adults, suggesting that the APOE ε4 status has limited sensitivity and its impact increases with fewer cognitive resources (i.e., with increasing impairment). Additionally, the impact of several genetic variants, including the APOE ε4 allele, on the brain and cognition may increase with age (Lindenberger et al., 2008), suggesting that there may be null effects among middle-aged adults but not older adults. Nevertheless, studies on cognitively healthy individuals ranging from late middle age to older adulthood have found smaller hippocampal volumes among ε4 carriers compared to non-carriers (den Heijer et al., 2002; Taylor et al., 2014) as have other studies with sample sizes comparable to the present study (e.g., Mueller et al., 2008).

The two ethnic groups included in the present study did differ neither in the hippocampal volumes nor in the gray matter volumes. Previous studies comparing hippocampal volumes between Hispanics/Latinos to non-Hispanic/Latino Whites have been mixed. DeCarli et al. (2008) and Mungas et al. (2009) consistently found similar hippocampal volumes among Hispanics/Latinos and non-Hispanic/Latino Whites, across their overlapping cohorts. In contrast, Zahodne et al. (2015) found smaller hippocampal volumes among Hispanics/Latinos relative to non-Hispanic/Latino Whites. Differences may be related to Hispanic/Latino country of origin differences. Hispanics/Latinos in DeCarli et al. (2008) and Mungas et al. (2009) studies tended to be of Mexican descent whereas the samples from Zahodne et al. (2015) tended to have greater proportions of Hispanics/Latinos of Caribbean descent. Although we do not have country of origin information on a significant proportion of the Hispanic/Latino participants, the data we have suggest that at least half of the sample was of Mexican descent.

### Regional Brain Volumes From Whole Brain Voxel-Wise Analysis

The whole brain voxel-based analysis determined that ε4 carriers had smaller volumes in the left superior temporal and the right middle temporal white matter but not the hippocampus. In particular, the left superior temporal white matter region appeared to be near the temporal parietal junction. The VBM findings may be reflective of damage to connections to-and-from the hippocampus prior to hippocampal structural damage. Temporal parietal areas have been shown to have reduced hypometabolism in AD (Alexander et al., 2002) and, in groups of both Hispanic/Latino and non-Hispanic/Latino White, cognitively healthy ε4 carriers compared to non-carriers (Reiman et al., 2005; Langbaum et al., 2010).

Consistent with the present findings, other voxel-based analyses did not find APOE ε4 status-related differences in the hippocampal regions (Wishart et al., 2006; ten Kate et al., 2016). Wishart et al. (2006) also found smaller volumes in the temporal regions (e.g., anterior regions of medial temporal lobe) but not in the hippocampus itself among a relatively younger sample (average age mid-50s) of ε4 carriers relative to non-carriers.

Regarding ethnicity, the comparison using VBM determined that non-Hispanic/Latino Whites had greater gray matter volumes in the left middle frontal gyrus. Longitudinal analysis is necessary to determine whether ethnic group differences in brain volumes translate to differential preservation of cognitive and functional resources later in life. Further, future studies should examine the APOE ε4 status in combination with other risk factors for AD that are more prevalent among Hispanics/Latinos (Haan et al., 2003), such as cardiovascular risk factors (Irie et al., 2008; Bangen et al., 2013; Willey et al., 2014). Hispanics/Latinos were no more or less likely to be impacted by APOE ε4 status than non-Hispanic/Latino Whites across cognitive or brain measures. The present sample is too small to compare various allelic presentations (ε3/ ε4 vs. ε2/ ε4), which may be important to consider when comparing ethnic groups. Maestre et al. (1995) found that the ε2 status was associated with an increased risk for AD after 70 years of age among Hispanics/Latinos and African Americans but not among non-Hispanic/Latino Whites. Haan et al. (2003) found that APOE ε4 homozygotes, but not ε4 heterozygotes, had increased the risk for dementia among a largely Mexican American group of Hispanics/Latinos, suggesting that these two groups may be more distinguished in Hispanics/Latinos than in non-Hispanic/Latino Whites. However, more recent data from the cohort suggest that over time and with older age, the impact of having one copy of the ε4 allele on incidence of MCI and dementia becomes stronger (Qian et al., 2017).

## Conclusion

Taken together, the present results highlight the need to identify group and individual differences in cognitive aging among Hispanics/Latinos in order to ameliorate health disparities in dementia.

## Data Availability Statement

Publicly available datasets were analyzed in this study. This data can be found at: https://www.alz.washington.edu/ and adni.loni.usc.edu.

## Ethics Statement

The studies involving human participants were reviewed and approved by institutions participating in the NACC (https://www.alz.washington.edu/) and ADNI (adni.loni.usc.edu) studies. The patients/participants provided their written informed consent to participate in this study.

## Author Contributions

AS contributed to project design, performed neuroimaging processing and analysis, ran statistical analysis, interpreted the data, and drafted the manuscript. AM contributed to neuroimaging and data analysis and critically reviewed the manuscript. SM contributed to neuroimaging processing and critically reviewed the manuscript. MG and JR contributed to project design and critically reviewed the manuscript. LR contributed to study design, data interpretation, and drafted and reviewed the manuscript. All authors contributed to the article and approved the submitted version.

## Conflict of Interest

The authors declare that the research was conducted in the absence of any commercial or financial relationships that could be construed as a potential conflict of interest.

## Footnotes

^1^https://www.alz.washington.edu/.

^2^adni.loni.usc.edu.

^3^For up-to-date information, see www.adni-info.org.

^4^http://www.adni-info.org/Scientists/CognitiveTesting.html.

^5^Specifics on NACC genotype and sequencing data can be found at the following (https://www.niagads.org/; https://alois.med.upenn.edu/adgc/about/overview.html).

^6^For more information on NACC and ADNI MRI procedures, see https://www.alz.washington.edu/WEB/mri_main.html and Jack et al. (2010; http://adni.loni.usc.edu/data-samples/mri/), respectively.

^7^https://surfer.nmr.mgh.harvard.edu/fswiki/.

^8^http://www.fil.ion.ucl.ac.uk/spm/.
